# Supplementary material for: Resurgent Na+ currents promote ultrafast spiking in projection neurons that drive fine motor control
Source: Nat Commun. 2021 Nov 19;12:6762. doi: 10.1038/s41467-021-26521-3 (PMC8604930; doi:10.1038/s41467-021-26521-3)
Supplement: Supplementary file 1 — Supplementary Information [file 41467_2021_26521_MOESM1_ESM.pdf]

Supplementary Information

Resurgent Na^+ currents promote ultrafast spiking in projection neurons that drive fine motor control

Benjamin M. Zemel[1], Alexander A. Nevue[2], Andre Dagostin[1], Peter V. Lovell[2], Claudio V. Mello[2], Henrique von Gersdorff[1,3]

[1]Vollum Institute, Oregon Health and Science University, Portland, Oregon 97239

[2]Department of Behavioral Neuroscience, Oregon Health and Science University, Portland, Oregon 97239

[3]Oregon Hearing Research Center, Oregon Health and Science University, Portland, Oregon 97239

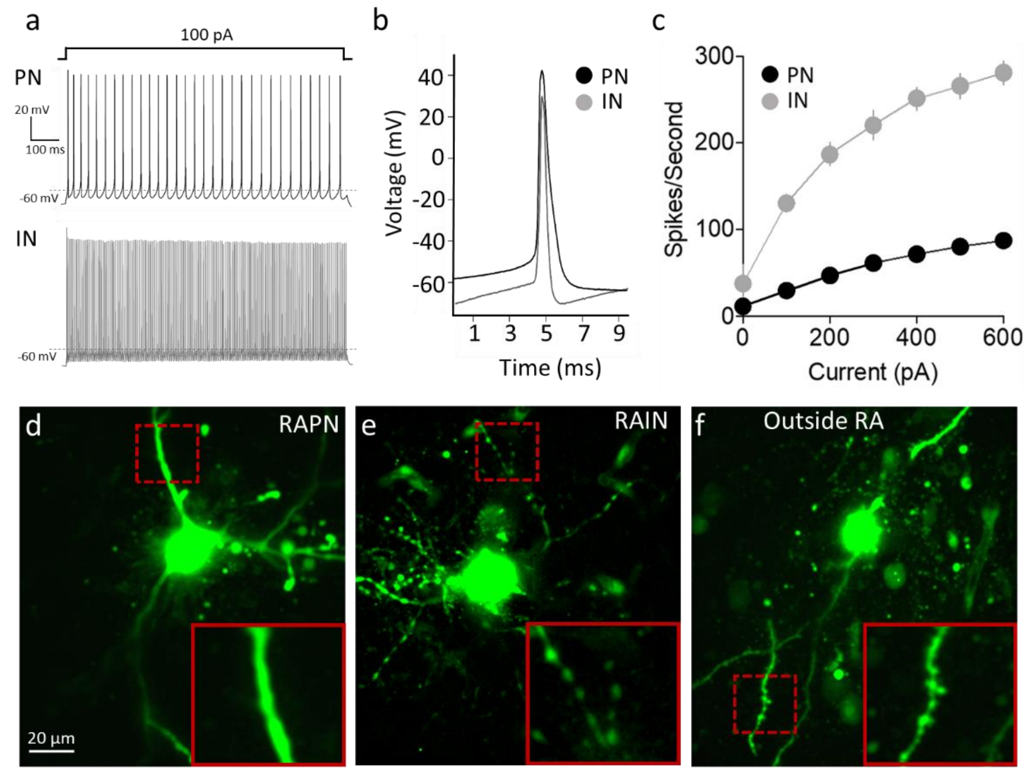

Supplementary Figure 1. Distinct morphology and excitable properties of arcopallial neurons in adult male zebra finches. a. AP trains elicited in a RAPN (projection neuron; top) and a RAIN (inhibitory interneuron; bottom) during a 1 sec 100 pA current injection at 25°C. **b.** Overlay of average from the first 5 APs in an RAPN and a RAIN (same cells as in a). **c.** Average elicited spikes/sec as a function of current injected in RAPNs and RAINs (N= 15 RAPNs and 4 RAINs). Data are presented as mean values +/- SEM. **d.** Representative image of a RA projection neuron (RAPN) that has been filled with a fluorescent dye (Alexafluor 488 hydrazide) during an electrophysiological recording. Note the lack of varicosities in its processes (dashed box expanded in the bottom right). Dye fills were performed for all voltage clamp experiments to confirm cell-type (N= 94 RAPNs). **e.** Representative image of an RA interneuron (RAIN) that has been filled with fluorescent dye as in (d). Note the prominent varicosities in its processes[24,39] (dashed box expanded in the bottom right). (N= 4 RAINs). **f.** Representative image of an arcopallial neuron outside RA (Outside RA) that has been filled with fluorescent dye as in (d-e). Note the dendritic processes with large spines that are distinct from RAPNs and INs (dashed box expanded in the bottom right). Dye fills were performed for all voltage clamp experiments to confirm cell-type (N= 11 neurons outside RA).

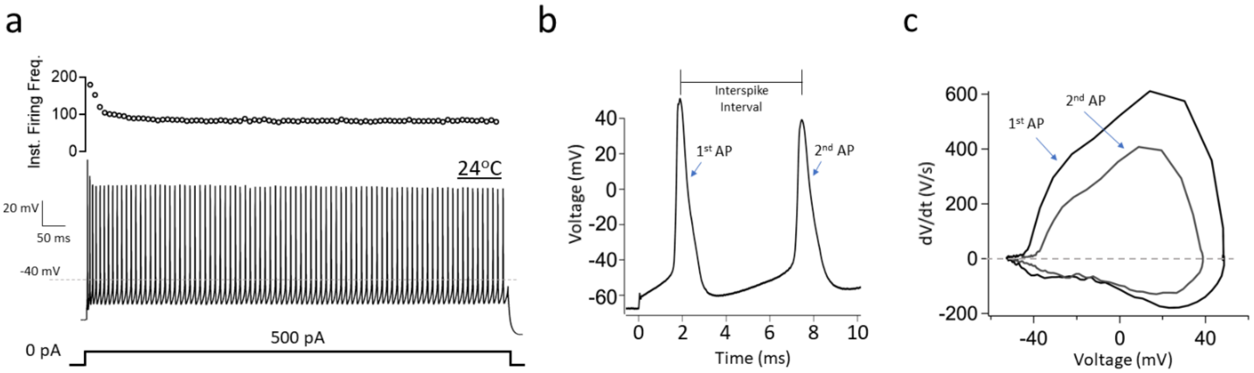

Supplementary Figure 2. Intrinsic excitable properties of RAPNs at room temperature. a. Representative AP train (from the same cell as in Fig. 1b) elicited during a 1 sec +500 pA current injection. Top: instantaneous firing frequency (Inst. Firing Freq., in Hz) plotted against time. **b.** First two APs from (a). **c.** Overlay of the phase plane plots from the two APs in (b).

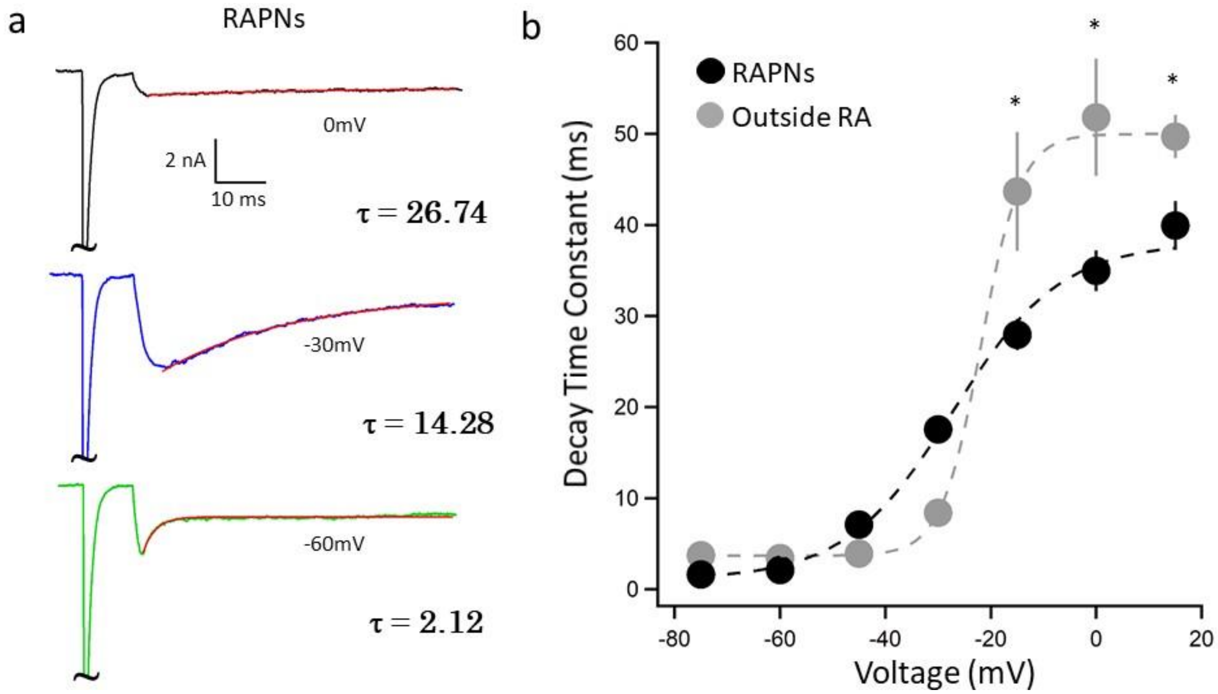

Supplementary Figure 3. Voltage-clamp recordings of arcopallial neurons in slices of adult male zebra finches. a. Individual current traces for multiple test potentials overlaid with single exponential decay fits (red trace) for I_{NaR} in RA projection neurons (RAPNs). I_{NaT} peaks have been truncated. **b.** I_{NaR} decay time constant as a function of test potential for RAPNs and neurons outside RA (Two-way ANOVA with Tukey's post hoc; $P = 5.4 \times 10^{-7}$, $F (6, 126) = 7.628$, N =12 RAPNs and 8 neurons outside RA; Individual comparisons: 15 mV (P = 0.04), 0 mV (P = 0.00003), -15 mV (P = 0.0001)). Data are presented as mean values +/- SEM. Stars depict significant age differences in RA determined by post hoc analyses; * = P ≤ 0.05.

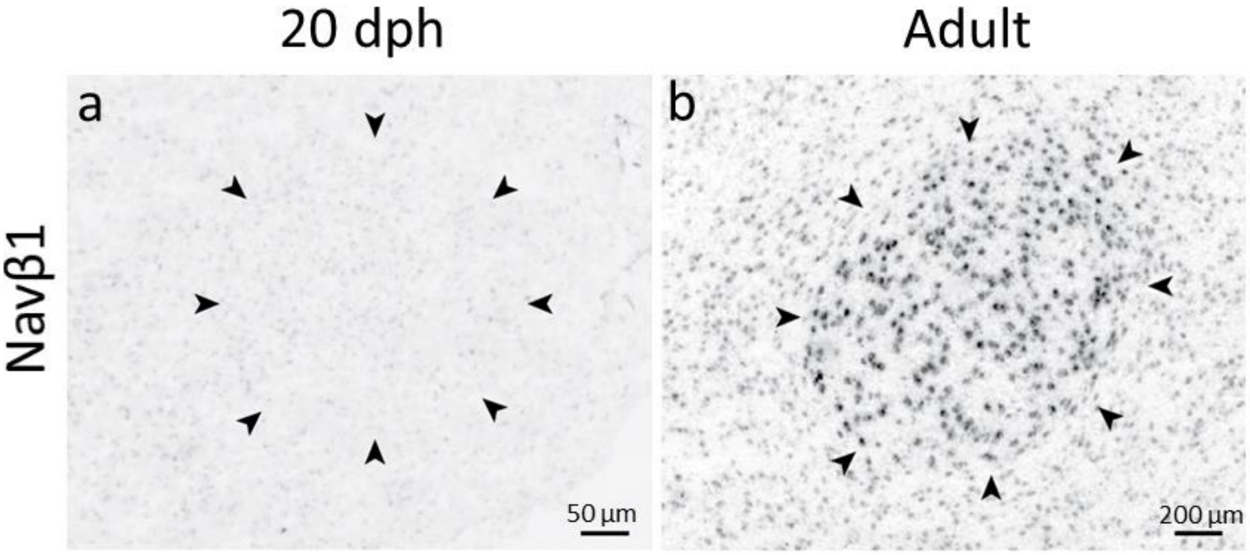

Supplementary Figure 4. Age-dependent change in expression of Navβ1 mRNA in the arcopallium of male zebra finches. a-b. Representative *in situ* hybridization images for Navβ1 mRNA in 20 dph and adult zebra finch arcopallium; black arrowheads indicate RA borders (N=3 birds per age; days post hatch, dph).

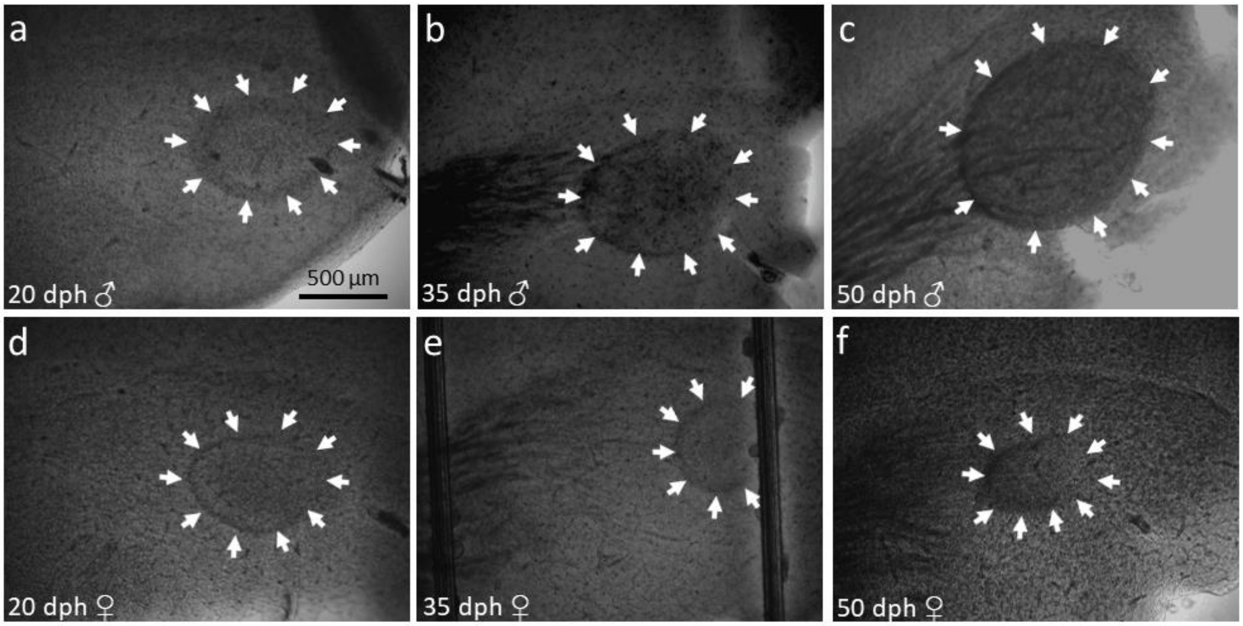

Supplementary Figure 5. IR-DIC visualization of RA in brain slices from zebra finches across ages. a-c. Representative images of parasagittal slices through the arcopallium of 20, 35, and 50 days post hatch (dph) males. RA was first identified under these conditions for all electrophysiological recordings. **d-f.** Representative images of parasagittal slices through the arcopallium of 20, 35, and 50 dph females. RA was first identified under these conditions for all electrophysiological recordings. In all panels, RA borders are indicated by white arrows. Note the increasing sex difference in RA size across ages, as well as the more pronounced myelination (dark fibers) in RA and surrounding arcopallium in males compared to females[61]. Dorsal is up and anterior to the left. Patterns were consistent across all animals.

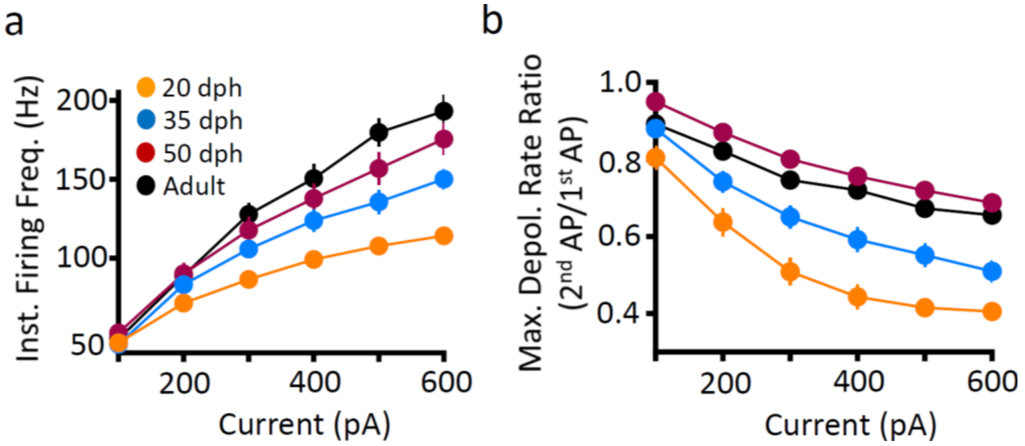

Supplementary Figure 6. Age-dependent changes in intrinsic excitable properties of RAPNs. a. Average instantaneous firing frequency (Inst. Firing Freq.) as a function of current injected for each age group (two-way ANOVA with Tukey's post hoc; P = 0.004, F (15, 361) = 2.320, N (cells/age) =18/20 dph, 20/35 dph, 20/50 dph and 14/adults). Data are presented as mean values +/- SEM. **b.** Fold-change of the maximum depolarization rate from the 1st to the 2nd AP (Max. Depol. Rate Ratio) as a function of current injected for each age group (two-way ANOVA with Tukey's post hoc; P = 7.1 x 10^{-8}, F (15, 348) = 6.774, N (cells/age) =18/20 dph, 20/35 dph, 20/50 dph and 14/adults). Data are presented as mean values +/- SEM.

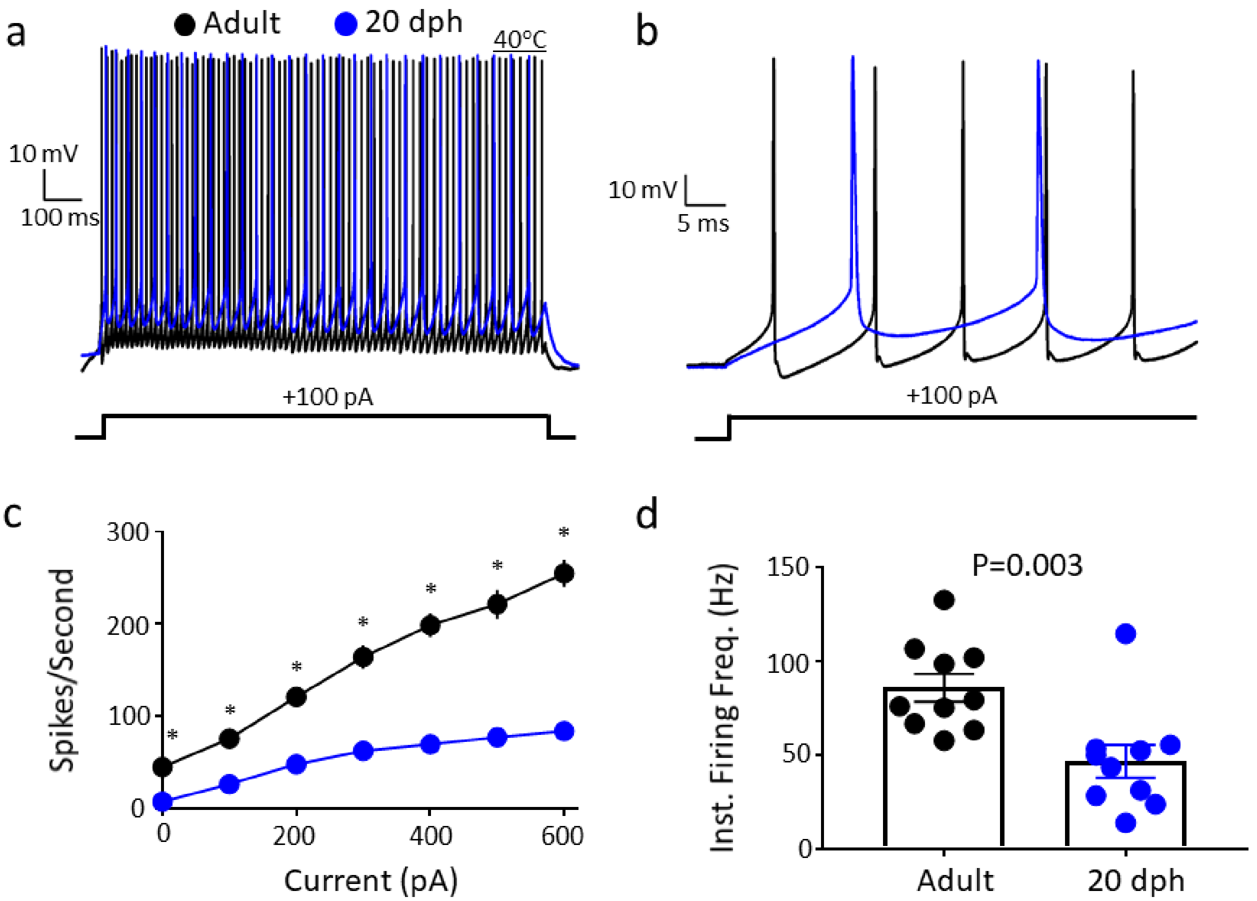

Supplementary Figure 7. Intrinsic excitable properties recorded in whole cell current clamp at 40°C in RAPNs of 20 days post hatch (dph) and adult male zebra finches. a. Overlay of APs (top) elicited by a +100 pA current injection (bottom) in 20 dph (blue) and adult (black) finches. **b.** Expanded x-axis from (a) showing the first few APs. **c.** Average number of spikes/sec as a function of current injected in 20 dph and adult males (two-way ANOVA with Tukey's post hoc; P = 1.9×10^{-14}, F (6, 111) = 17.94, N (cells/age) = 8/adult and 10/20 dph finches; Individual comparisons: 0 pA (P = 0.002), 100 pA (P = 0.0002), 200 pA (P = 3.2×10^{-8}), 300 pA (P = 8.6×10^{-14}), 400 pA (P = 4.2×10^{-20}), 500 pA (P = 3.7×10^{-23}), 600 pA (P = 3.7×10^{-27})). Data are presented as mean values +/- SEM. Stars depict significant age differences in RA determined by post hoc analyses; * = P ≤ 0.05. **d.** Average instantaneous firing frequency measured in response to a +100 pA current injection in 20 dph and adult males; N (cells/age) = 10/20 dph and 10/adults; two-tailed Student's t-test. Data are presented as individual data points with bars as mean values +/- SEM.

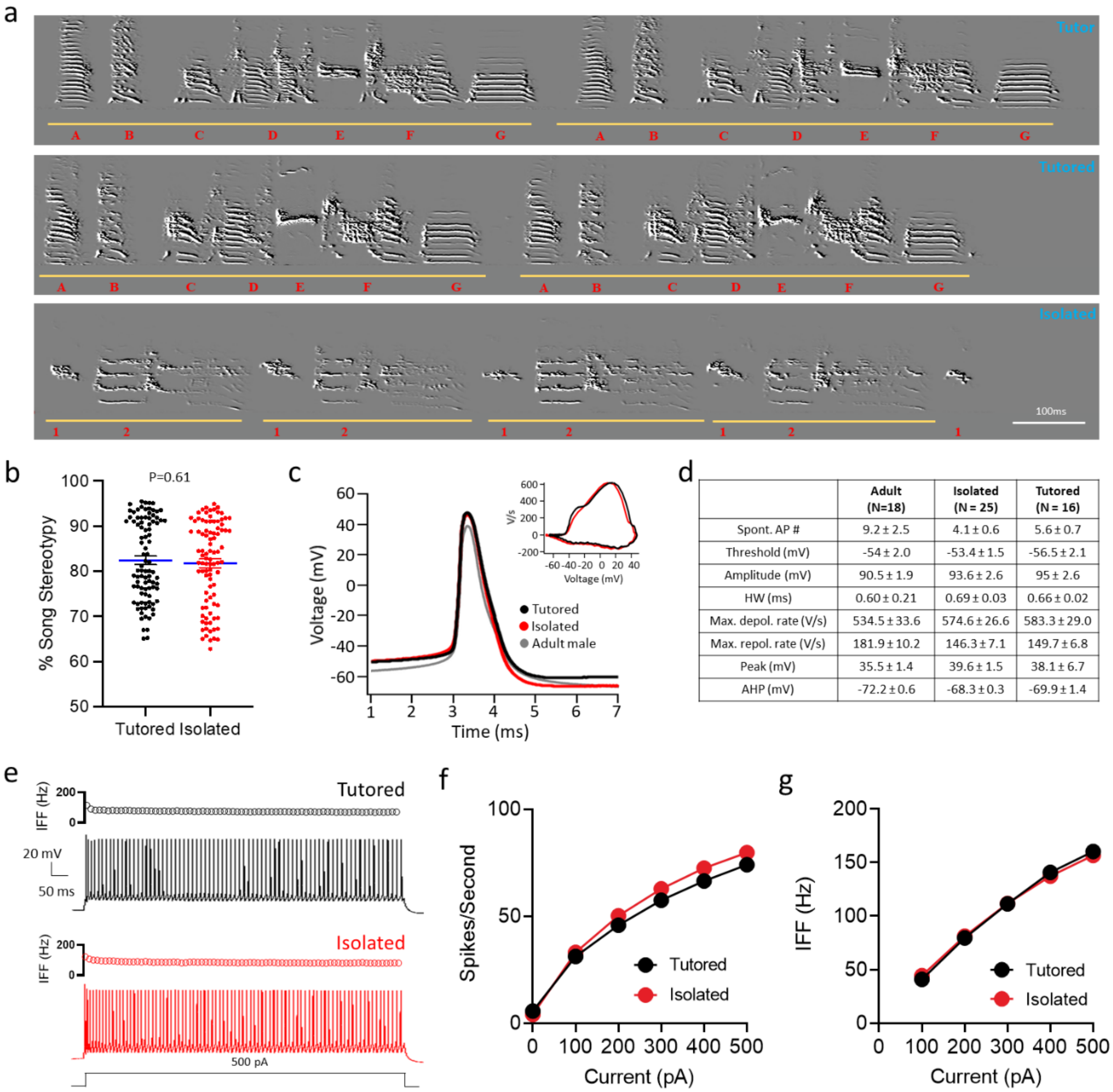

	Adult (N=18)	Isolated (N = 25)	Tutored (N = 16)
Spont. AP #	9.2 ± 2.5	4.1 ± 0.6	5.6 ± 0.7
Threshold (mV)	-54 ± 2.0	-53.4 ± 1.5	-56.5 ± 2.1
Amplitude (mV)	90.5 ± 1.9	93.6 ± 2.6	95 ± 2.6
HW (ms)	0.60 ± 0.21	0.69 ± 0.03	0.66 ± 0.02
Max. depol. rate (V/s)	534.5 ± 33.6	574.6 ± 26.6	583.3 ± 29.0
Max. repol. rate (V/s)	181.9 ± 10.2	146.3 ± 7.1	149.7 ± 6.8
Peak (mV)	35.5 ± 1.4	39.6 ± 1.5	38.1 ± 6.7
AHP (mV)	-72.2 ± 0.6	-68.3 ± 0.3	-69.9 ± 1.4

Supplementary Figure 8. Song analysis and the comparison of excitable properties of RAPNs from socially isolated and normally reared (tutored) finches. a. Representative examples of sonograms of female directed song obtained from a single clutch containing a tutor (adult; top), tutored bird (65 dph male; middle) and isolated sibling (65 dph; bottom). **b.** Individual value plot of % stereotypy values calculated for unidirectional pairwise comparisons of song motifs sung by individual tutored (n=2) or isolated (n=2) birds (two-tailed Student's t-test; P = 0.41). Data are presented as mean (blue) values +/- SEM. **c.** Overlay of averaged spontaneous APs recorded in individual RAPNs from a 65 dph tutored bird (black) and its isolated sibling (red). Adult male AP included for reference (grey). Inset is an overlay of AP phase plane plots derived from the 65 dph birds. **d.** Table with summary data from spontaneous APs recorded in tutored and isolated

65 dph male finches (comparison of all parameters; pairwise Student's T-test; P>0.05). Adult male data included for reference. Data shown as Mean ± SE. Spont. AP # = number of spontaneous APs produced in 1 sec, HW = half-width, Max. depol. Rate = Maximum depolarization rate, Max. repol. rate = Maximum repolarization rate. **e.** Representative AP trains elicited by a 1 sec +500 pA current injection in RAPNs from tutored (black, top) and isolated (red, bottom) siblings in (c).The corresponding plot of firing frequency as function of time is shown at the top of each train. **f.** Average number of spikes produced during 1 sec as a function of current injected for each group (two-way ANOVA; P = 0.34,F (5, 230) = 1.190, N(cells) =15 tutored and 25 isolated). **g.** Average instantaneous firing frequency (IFF, in Hz) as a function of current injected for each group (two-way ANOVA; P = 0.90, F (4, 190) = 2.906; N(cells) =15 tutored and 25 isolated).

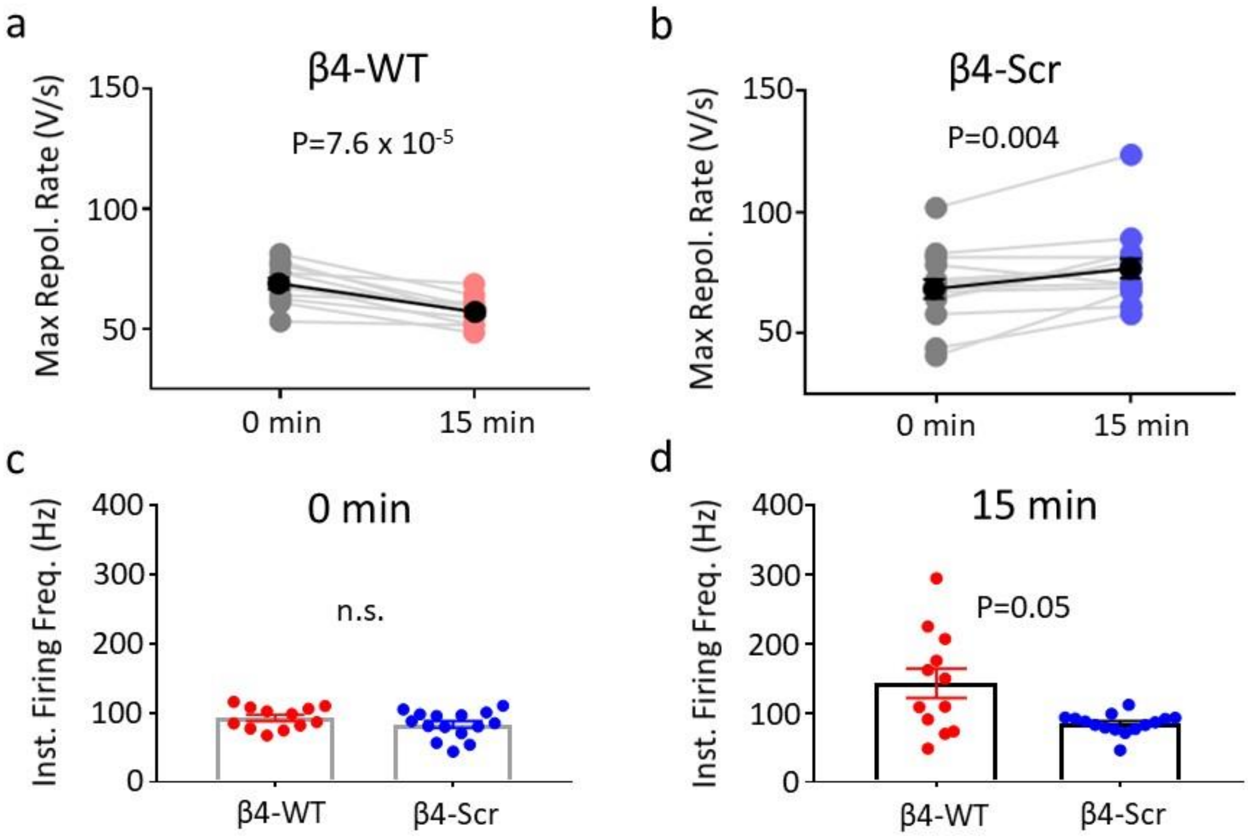

Supplementary Figure 9. Effects of Navβ4 C-terminal peptide (β4-WT) on intrinsic excitable properties of RAPNs in 20 dph males. a-b. Time-dependent effects of β4-WT or β4-Scr on the maximum repolarization rate (Max. Repol. Rate) in individual cells (mean ± SEM in black; two-tailed paired t-test). **c-d.** Comparisons of instantaneous firing frequencies (Inst. Firing Freq.) of the first 2 APs elicited immediately or 15 min after achieving the whole-cell configuration between cells exposed to β4-WT (red) or β4-Scr (blue); two-tailed Student's t-test in (c) and Mann-Whitney test in (d). N= 12 and 15 cells recorded with β4-WT and β4-Scr respectively. Data are presented as individual data points with bars as mean values +/- SEM.

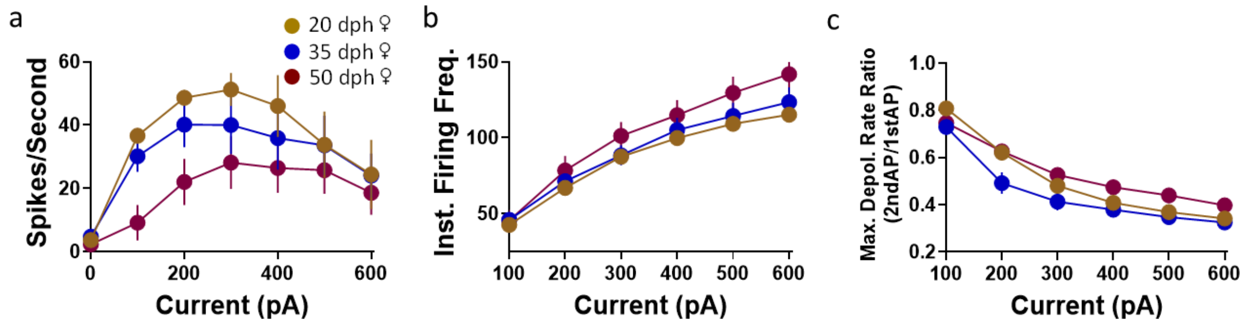

Supplementary Figure 10. Intrinsic excitable properties of RAPNs in female zebra finches across ages. a. Average number of spikes/sec as a function of current injected for each age group. Two-way ANOVA with Tukey's post hoc; P = 0.77, F (12, 296) = 0.6816, N (cells/age) = 21/20 dph, 23/35 dph, 7/50 dph and 18/adult cells. Data are presented as mean values +/- SEM. **b.** Average instantaneous firing frequency (Inst. Firing Freq., in Hz) measured as a function of current injected for each age group. Two-way ANOVA with Tukey's post hoc; P = 0.99 , F (10, 211) = 0.3687, N (cells/age) = 21/20 dph, 23/35 dph, 7/50 dph and 18/adult cells. Data are presented as mean values +/- SEM. **c.** Fold-change of the maximum depolarization rate from the first to the second AP (Max. Depol. Rate Ratio) as a function of current injected for each age group. Two-way ANOVA with Tukey's post hoc; P = 0.51 , F (10, 207) = 0.9226; N (cells/age) = 21/20 dph, 23/35 dph, 7/50 dph and 18/adult cells. Data are presented as mean values +/- SEM.

a

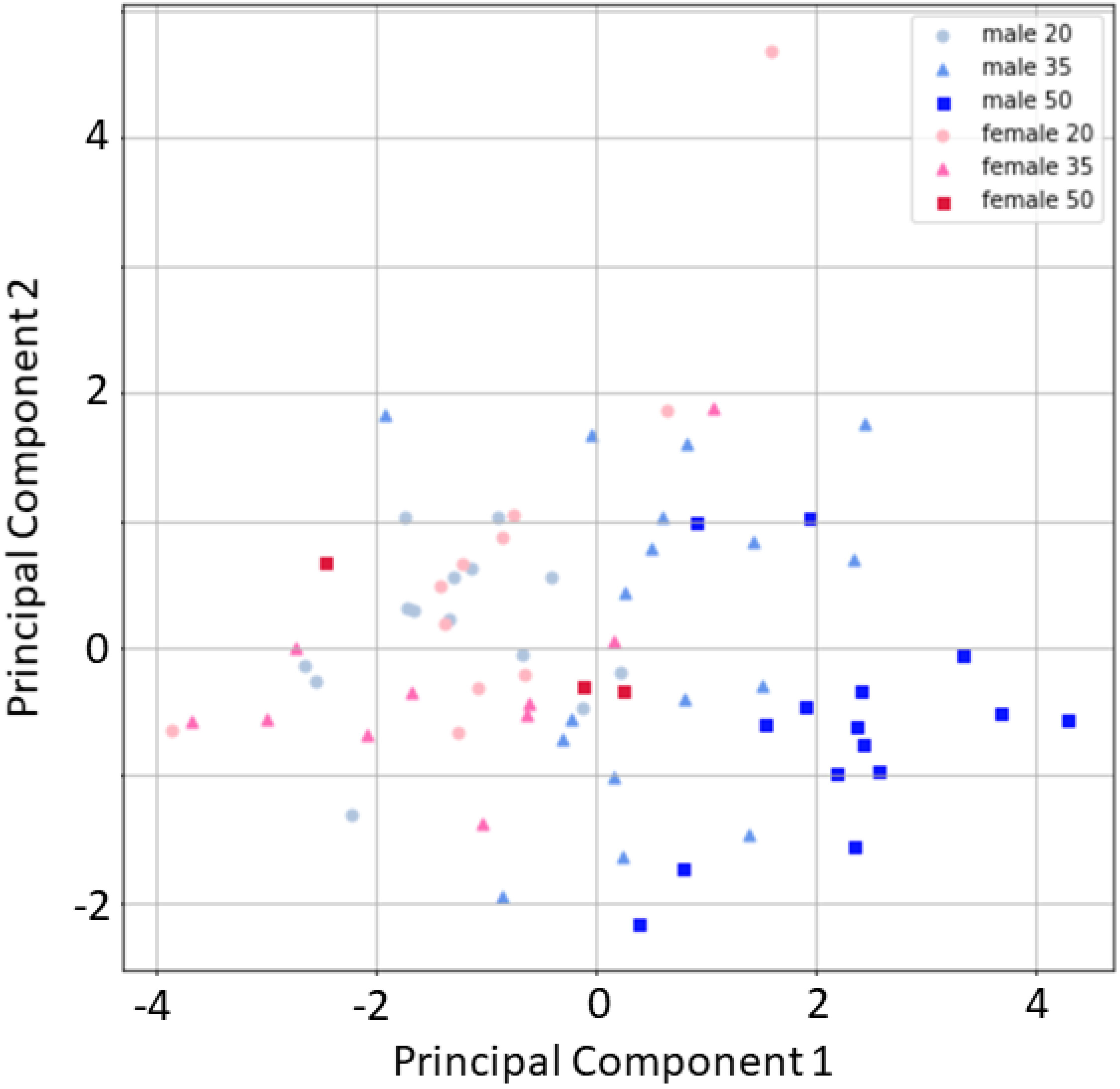

Supplementary Figure 11. Principle component analysis (PCA) for spontaneous APs in female and male finches across development. a. PCA of spontaneous spike frequency, threshold, half-width, maximum depolarization rate and maximum repolarization rate in males (blue) and females (red), different color ages represented by different shapes and color shades (days post hatch indicated in the legend). PC1 and PC2 accounted for 53% and 20%, respectively, of the variance across ages and sex.
