# Supplementary material for: Resurgent Na+ currents promote ultrafast spiking in projection neurons that drive fine motor control
Source: Nat Commun. 2021 Nov 19;12:6762. doi: 10.1038/s41467-021-26521-3 (PMC8604930; doi:10.1038/s41467-021-26521-3)
Supplement: Supplementary file 3 — Reporting Summary [file 41467_2021_26521_MOESM3_ESM.pdf]

nature research

Corresponding author(s): Henrique von Gersdorff

Last updated by author(s): Sep 20, 2021

Reporting Summary

Nature Research wishes to improve the reproducibility of the work that we publish. This form provides structure for consistency and transparency in reporting. For further information on Nature Research policies, see our Editorial Policies and the Editorial Policy Checklist.

Statistics

For all statistical analyses, confirm that the following items are present in the figure legend, table legend, main text, or Methods section.

n/a	Confirmed	
☐	☒	The exact sample size (*n*) for each experimental group/condition, given as a discrete number and unit of measurement
☐	☒	A statement on whether measurements were taken from distinct samples or whether the same sample was measured repeatedly
☐	☒	The statistical test(s) used AND whether they are one- or two-sided<br>*Only common tests should be described solely by name; describe more complex techniques in the Methods section.*
☐	☒	A description of all covariates tested
☐	☒	A description of any assumptions or corrections, such as tests of normality and adjustment for multiple comparisons
☐	☒	A full description of the statistical parameters including central tendency (e.g. means) or other basic estimates (e.g. regression coefficient) AND variation (e.g. standard deviation) or associated estimates of uncertainty (e.g. confidence intervals)
☐	☒	For null hypothesis testing, the test statistic (e.g. *F*, *t*, *r*) with confidence intervals, effect sizes, degrees of freedom and *P* value noted<br>*Give P values as exact values whenever suitable.*
☐	☒	For Bayesian analysis, information on the choice of priors and Markov chain Monte Carlo settings
☐	☒	For hierarchical and complex designs, identification of the appropriate level for tests and full reporting of outcomes
☒	☐	Estimates of effect sizes (e.g. Cohen's *d*, Pearson's *r*), indicating how they were calculated

Our web collection on statistics for biologists contains articles on many of the points above.

Software and code

Policy information about availability of computer code

Data collection	Gene expression data from in situ hybridization experiments were collected using the open source FIJI version of ImageJ v2.0.0-rc-69/1.52p (Imaged on Neurolucida 16). Songs were collected using Sound Analysis Pro (SAP) software v2011.087 (soundanaylsispro.com). Electrophysiology data (voltage- and current-clamp) were collected using HEKA EPC-10/2 amplifier controlled by Patchmaster software v2x91 (HEKA). Data from dynamic clamp experiments were collected using custom macros that were written in Igor Pro v8.0 (Wavemetrics) that interfaced with the HEKA EPC-10/2 amplifier (HEKA) through a National Instruments DAQ board (PCIe-6321) controlled by Igor v8.0 using NIDAQ Tools MX package (Wavemetrics). A complete description of the code used in the dynamic clamp experiments is included with this Reporting Summary. The new code used for dynamic clamp experiments can be found at the Senselab database (ModelDB, accession #: 267132).
Data analysis	Electrophysiology data were analyzed using standard macros available with Igor Pro v8.0 (Wavemetrics). Electrophysiology and gene expression data were further analyzed in Excel (Microsoft Office 2016), and statistical analyses were performed in Prism v8.3.0 (GraphPad) and the Anaconda v1.9.7 distribution of Python v3.4 with a Jupyter environment v6.0.1. Songs were analyzed using Sound Analysis Pro (SAP) software v2011.087 (soundanaylsispro.com) and Raven Lite v2.0.1 (ravensoundsoftware.com).

For manuscripts utilizing custom algorithms or software that are central to the research but not yet described in published literature, software must be made available to editors and reviewers. We strongly encourage code deposition in a community repository (e.g. GitHub). See the Nature Research guidelines for submitting code & software for further information.

Data

Policy information about availability of data

All manuscripts must include a data availability statement. This statement should provide the following information, where applicable:

- Accession codes, unique identifiers, or web links for publicly available datasets
- A list of figures that have associated raw data
- A description of any restrictions on data availability

Results and discussion referencing the zebra finch atlas and in situ hybridizations from adult male finches can be found at the Zebra Finch Expression Brain Atlas (zebrafinchatlas.org). Previously published code that was used as a template for dynamic clamp experiments can be found at ModelDB within the Senselab database (see accession number 127021). The new code used for dynamic clamp experiments can be found at the Senselab database (ModelDB, accession #: 267132). Please refer to the Source Code in the Data Availability Statement for the datasets generated and/or analyzed during the current study from Main Figures (2-10) and Supplementary Figures (1,3 and 6-11).

Field-specific reporting

Please select the one below that is the best fit for your research. If you are not sure, read the appropriate sections before making your selection.

☒ Life sciences ☐ Behavioural & social sciences ☐ Ecological, evolutionary & environmental sciences

For a reference copy of the document with all sections, see nature.com/documents/nr-reporting-summary-flat.pdf

Life sciences study design

All studies must disclose on these points even when the disclosure is negative.

Sample size	All sample sizes represent the minimum sample size for detecting age and/or sex group differences in gene expression analysis (i.e. cell counts and/or brain expression levels; Mello and Clayton, 1994; Mello et al., 1992), or patch-clamp electrophysiological characterization of properties within a single vocal nucleus (Dutar and Perkel, 1998).
Data exclusions	No data was excluded from analysis.
Replication	To control for genetic difference in non-inbred zebra finches, juvenile finches were obtained from several different breeding clutches, and randomly assigned to different age and/or sex groups that were then analyzed for group differences in gene expression and electrophysiology properties as described in detail in the methods section. By obtaining same-age birds from different clutches we could determine the distribution of the data between individuals to verify the reproducibility of our experimental findings. There were no findings that we could identify that could not be replicated in this study.
Randomization	Zebra finches were allocated into experimental groups based on 1) age and 2) sex.
Blinding	Blinding only occurred when birds were selected, at random, from the their housing at specific ages for electrophysiological and molecular studies. Differences in plumage and/or RA size between 1)males vs. females, 2)adult vs. juveniles and 3) isolates vs. controls did not allow for blinding in these studies for the experimenter sacrificing and performing experiments with animal tissue.

Reporting for specific materials, systems and methods

We require information from authors about some types of materials, experimental systems and methods used in many studies. Here, indicate whether each material, system or method listed is relevant to your study. If you are not sure if a list item applies to your research, read the appropriate section before selecting a response.

Materials & experimental systems

n/a	Involved in the study	
☐	☒	Antibodies
☒	☐	Eukaryotic cell lines
☒	☐	Palaeontology and archaeology
☐	☒	Animals and other organisms
☒	☐	Human research participants
☒	☐	Clinical data
☒	☐	Dual use research of concern

Methods

n/a	Involved in the study	
☒	☐	ChIP-seq
☒	☐	Flow cytometry
☒	☐	MRI-based neuroimaging

Antibodies

Antibodies used	DIG antibodies (Roche #110932749100)

Validation	Antibodies were validated by the manufacturer (Roche #110932749100) that states "no cross-reactivity with other steroids, such as human estrogens and androgens." Additional validation of anti-body specificity for use in in situ hybridization experiments is determined in the following publication: Carleton, J. B., Lovell, P. V., McHugh, A., Marzulla, T., Horback, K. L., & Mello, C. V. (2014). An optimized protocol for high-throughput in situ hybridization of zebra finch brain. Cold Spring Harbor Protocols, 2014(12), pdb-prot084582.

Animals and other organisms

Policy information about studies involving animals; ARRIVE guidelines recommended for reporting animal research

Laboratory animals	Zebra Finch (Taeniopygia guttata), Male and Female, Laboratory animals (not wild)
Wild animals	No wild animals were used in this study
Field-collected samples	No field-collected samples were used in this study
Ethics oversight	Institutional Animal Care and Use Committee (IACUC) at Oregon Health and Science University, American Association for Accreditation of Laboratory Animal Care (AAALAC) at Oregon Health and Science University

Note that full information on the approval of the study protocol must also be provided in the manuscript.
